# Analysis of the measurements used as potency tests for the 31 US FDA-approved cell therapy products

**DOI:** 10.1186/s12967-025-06253-4

**Published:** 2025-03-04

**Authors:** Carl G. Simon, Erich H. Bozenhardt, Christina M. Celluzzi, David Dobnik, Melanie L. Grant, Uma Lakshmipathy, Thiana Nebel, Linda Peltier, Anthony Ratcliffe, James L. Sherley, Glyn N. Stacey, Rouzbeh R. Taghizadeh, Eddie H. P. Tan, Sandrine Vessillier

**Affiliations:** 1 Biosystems & Biomaterials Division, National Institute of Standards & Technology (NIST), Gaithersburg, MD USA; 2 United Therapeutics Corporation, Regenerative Medicine Operations, Research Triangle Park, Durham, NC USA; 3 Association for the Advancement of Blood & Biotherapies (AABB), Bethesda, MD USA; 4 Niba Labs, Ljubljana, Slovenia; 5 National Institute of Biology, Ljubljana, Slovenia; 6 Department of Pediatrics, Marcus Center for Cellular & Gene Therapies, Correlative Studies Laboratory, Emory University School of Medicine, Children’s Healthcare of Atlanta, Atlanta, GA USA; 7 Pharma Services, Science & Technology, Thermo Fisher Scientific, San Diego, CA USA; 8 Medical Education, Sports Medicine & Orthobiologics, Medical Sales Institute, San Diego, CA USA; 9 Cellular Therapy Lab, Research Institute of McGill University Health Center, Montreal, QC Canada; 10 Synthasome, Inc, San Diego, CA USA; 11 Asymmetrex, LLC, Boston, MA USA; 12 International Stem Cell Biobanking Initiative, Barley, Herts UK; 13 National Stem Cell Resource Centre, Institute of Zoology, Chinese Academy of Sciences, Beijing, China; 14 Beijing Institute for Stem Cells and Regenerative Medicine, Chinese Academy of Sciences, Beijing, China; 15 Kendall Innovations, Cambridge, MA USA; 16 Centre of Regulatory Excellence, Duke-NUS Medical School, Singapore, Singapore; 17 Science, Research and Innovation Group - Biotherapeutics and Advanced Therapies Division, Medicines and Healthcare Products Regulatory Agency, South Mimms, Hertfordshire, UK

**Keywords:** Cell therapy product, Potency, Potency test, Regenerative medicine

## Abstract

Cell therapy product (CTP) developers face the significant challenge of developing appropriate potency tests for their CTPs. A review of the known potency tests used for the 31 United States Food and Drug Administration-approved CTPs (US FDA) can guide developers in designing effective potency tests for future CTPs. Data on these tests were primarily collected from publicly available regulatory documentation on the US FDA website (90%) as well as other sources (literature, company communications, etc.). Based on these data, an estimated 104 total potency tests have been used for the 31 CTPs. Of these, 33 are redacted (32%), leaving 71 non-redacted potency tests. On average, each CTP has 3.4 potency tests (standard deviation 2.0). The 71 non-redacted potency tests were categorized into 5 bins: “Viability and count” (37 tests, 52%), “Expression” (19 tests, 27%), “Bioassays” (7 tests, 7%), “Genetic modification” (6 tests, 9%) and “Histology” (2 tests, 3%). Measurements of gene or protein expression were used by 20 of the 31 CTPs (65%), and 19 CTPs (61%) used measurements of cell viability or cell count as a potency test. “Viability and count” and “Expression” are the two tests that have most often been used together for the same product, occurring for 16 CTPs (52%). It is unclear if bioassays are commonly used as potency tests since only 7 of 31 CTPs (23%) reported bioassays as potency tests. However, due to redactions, as many 24 (77%) CTPs could potentially have a bioassay as a potency test. Additionally, 26 of the 31 CTPs (84%) cite physicochemical assays (non-bioassays) as a potency test. This analysis of potency tests for approved CTPs provides valuable insights for developing potency tests for new CTPs.

**Supplementary Information:**

The online version contains supplementary material available at 10.1186/s12967-025-06253-4.

## Background

Developing suitable potency tests remains a significant challenge for cell therapy products (CTPs) [1–8]. Many of the issues and discussions surrounding CTP potency tests are summarized in a previous article [8]. Other examples include the FDA Advisory Committee Meeting Review of Mesoblast’s remestemcel-L in 2020 [9] and the FDA approval documentation for Iovance’s Amtagvi [10]. The previous article also proposes a potentially useful framework for understanding the relationships between mechanism of action, potency and efficacy [8]. This current perspective reviews and analyzes the types of measurements that have been used for release testing as potency tests for the 31 US FDA-approved CTPs for the time span of 2010 through 2024. US regulations require that CTPs, and all products regulated as biologics, have a potency test that is used for release testing for licensure [11]. Typically, the potency test is a release test performed on the final manufactured product after packaging. The goal of the potency test is to assure that the product can achieve its intended mechanism of action, to assess manufacturing consistency and to evaluate product stability [12, 13]. Potency tests may also be used for process design, manufacturing process control and in-process testing [13]. This article focuses on potency tests used as lot release tests. These analyses can help CTP developers identify suitable potency test measurements for new CTPs.

### Sources of information

Seven sources of information were used for this analysis. The primary source is the FDA website, “Approved Cellular and Gene Therapy Products”, which provides a key document for each CTP called the “Summary Basis for Regulatory Action” (SBRA) [14]. The SBRA lists the potency tests used for each CTP. SBRAs accounted for more than 90% of the information in this report. Additional sources include two slide decks from FDA advisory committee meetings [15, 16], a peer-reviewed literature report [17], a European Medical Association Assessment Report [18], a company’s referral to a poster abstract [19] and an FDA document entitled “Clinical and Clinical Pharmacology Review and Evaluation” [20]. Table 1 summarizes the available CTP potency test information used for analysis.

**Table 1:** Potency Tests Used for the 31 US FDA-Approved Cell Therapy Products (to date)

Product (Year Approved)	Description	Potency Tests
Hemacord (2011)	Allogeneic cord blood for hematopoietic and immunologic reconstitution in patients with disorders affecting the hematopoietic system that are inherited, acquired, or result from myeloablative treatment	FDA SBRA [14]: (i) Total nucleated cells (TNC); (ii) viability of CD45 + cells; (iii) viable CD34 + cell count; (iv) Colony forming unit (CFU)
Clinimmune (2012)		FDA SBRA: (i) Total nucleated cells (TNC); (ii) viability of total nucleated cells (TNC); (iii) viable CD34 + cell count; (iv) redacted
Ducord (2012)		FDA SBRA: (i) Total nucleated cells (TNC); (ii) viable nucleated cells; (iii) viable CD34 + cells (flow cytometry); (iv) redacted; (v) redacted
Lifesouth (2013)		FDA SBRA: (i) Total nucleated cells (TNC); (ii) viable nucleated cells; (iii) viable CD34 + cells (flow cytometry); (iv) redacted
Bloodworks (2016)		FDA SBRA: (i) Total nucleated cells (TNC); (ii) viable nucleated cells; (iii) viable CD34 + cells (flow cytometry); (iv) redacted
Allocord (2016)		FDA SBRA: (i) Total nucleated cells (TNC); (ii) viable nucleated cells; (iii) viable CD34 + cell count; (iv) colony forming units (CFU)
Clevecord (2016)		FDA SBRA: (i) Total nucleated cell number; (ii) viability of TNC; (iii) viable CD34 + cell count; (iv) redacted
MD Anderson (2018)		FDA SBRA: (i) Total CD34 + count; (ii) total nucleated cell (TNC) count (per HPC, cord blood); (iii) nucleated RBC; (iv) viability of nucleated cells; (v) viable CD34 + cells; (vi) colony forming unit (CFU) assay
Omisirge (2023)	Allogeneic umbilical cord blood (UCB) cells cultured in nicotinamide to improve stemness for patients with hematologic malignancies having UCB transplantation	FDA SBRA: CD34 + cell fold-increase
Kymriah (2017)	Autologous T-cells reprogrammed to target cells that express specific surface antigens for treating cancers (CAR T-cells)	FDA Advisory Committee Meeting [15]: i) CAR expression by flow cytometry; (ii) release of IFNγ in response to CD19-expressing target cells
Yescarta (2017)		FDA SBRA: (i) Cell viability; (ii) anti-CD19 CAR expression; (iii) redacted; Papadouli et al., 2020*:* Interferon-γ production by product upon stimulation with CD19 + cells [17]
Tecartus (2020)		FDA SBRA: (i) Cell viability; (ii) anti-CD19 CAR expression; (iii) redacted
Breyanzi (2021)		FDA SBRA: (i) Redacted
Abecma (2021)		FDA SBRA: (i) Redacted; EMA Assessment Report [18]: Interferon-γ production by product upon stimulation with BCMA + cells
Carvykti (2023)		FDA SBRA: (i) CAR expression from viable T cells; (ii) redacted
Aucatzyl (2024)		FDA SBRA: (i) Redacted
Tecelra (2024)	Autologous T cells genetically modified with lentiviral vector to express a T cell receptor (TCR) specific for human MAGE-A4 for treating synovial sarcoma	FDA SBRA: (i) Cytotoxic activity (cytotoxicity assay with flow cytometry)
Provenge (2010)	Autologous CD54 + cells activated with PAP-GM-CSF for prostate cancer	FDA SBRA: (i) Number of CD54 + cells (flow cytometry); (ii) increased expression of CD54 on the surface of antigen presenting cells after culture with PAP-GM-CSF (flow cytometry)
Laviv (2011)	Autologous fibroblasts from skin punch biopsy for nasolabial fold wrinkles	FDA SBRA: (i) Cell count; (ii) cell viability; (iii) collagen production by the cells
Gintuit (2012)	Allogeneic cultured keratinocytes and fibroblasts in bovine collagen for gingival defects	FDA SBRA: Histology with morphological assessments: epidermal coverage, epidermal development, basal cell layer keratinocyte viability, suprabasal cell layer keratinocyte viability, dermal thickness, fibroblast density, and matrix integrity
MACI (2016)	Autologous cultured chondrocytes on porcine collagen membrane for knee cartilage defects	FDA SBRA: (i) Cell number; (ii) redacted; (iii) redacted; Rapko et al. [19]: PCR measurement of aggrecan gene expression
Stratagraft (2021)	Allogeneic cultured keratinocytes and dermal fibroblasts in murine collagen for thermal burns	FDA SBRA: Redacted
Rethymic (2021)	Allogeneic processed thymus tissue for athymia	FDA SBRA: Histology-based (tissue organization, viability & retention of important cell types believed to be important for function)
Zynteglo (2022)	Autologous CD34 + cells hematopoietic stem cells (HSCs) transduced to express βA-T87Q-globin for ß-thalassemia	FDA SBRA: (i) Vector copy number (VCN) (qPCR); (ii) percent LVV + cells; (iii) redacted; (iv) redacted; (v) colony forming cells (CFC); (vi) βA-T87Q-globin quantitative protein expression
Skysona (2022)	Autologous CD34 + hematopoietic stem cells (HSCs) transduced with adrenoleukodystrophy protein (ALDP) for cerebral adrenoleukodystrophy (CALD)	FDA SBRA: (i) Vector copy number (VCN) (qPCR); (ii) percent LVV + cells; (iii) redacted; (iv) redacted; (v) redacted; (vi) percent ALDP + cells
Lantidra (2023)	Allogeneic pancreatic islet cellular therapy for Type 1 diabetes	FDA Advisory Committee Meeting [16]: (i) Glucose Stimulation Index (GSI): ELISA (enzyme linked immunosorbent assay) quantification of insulin release by glucose stimulated islets; (ii) Islet Yield: Dithizone (DTZ) stain and microscopic quantification; (iii) Viability: SYTO 13 green/ethidium bromide staining and microscopic evaluation
Casgevy (2023)	Autologous CD34 + HSCs gene edited to reduce BCL11A expression in erythroid lineage cells for treating ß-thalassemia in children	FDA SBRA: (i) On-target editing frequency (tracking of indels by decomposition, TIDE); (ii) redacted; (iii) redacted
Lyfgenia (2023)	Autologous HSCs containing functional copies of a modified β-globin gene for sickle cell disease	FDA SBRA: (i) Vector copy number (VCN); (ii) redacted, (iii) redacted; (iv) redacted; (v) redacted; (vi) βA-T87Q-globin quantitative protein expression
Amtagvi (2024)	Autologous T-cell therapy made of ex vivo-expanded lymphocytes harvested from patient tumors for melanoma	FDA SBRA: (i) redacted, (ii) redacted, (iii) redacted, (iv) redacted, (v) dose (total viable cells), (vi) redacted and (vii) redacted
Lenmeldy (2024)	Autologous HSCs expressing the human arylsulfatase (ARSA) gene for treating metachromatic leukodystrophy in children	FDA SBRA: (i) viability (%); (ii) vector copy number; (iii) vector copy number (calculation); (iv) transduction efficiency; (v) transgene function (arylsulfatase A (ARSA) activity); (vi) redacted; (vii) redacted; (viii) redacted
Ryoncil (2024)	Allogeneic culture-expanded MSCs isolated from the bone marrow of healthy human adult donors	FDA SBRA: (i) interleukin-2 receptor alpha (IL-2Rα) inhibition bioassay [20]; (ii) redacted; (iii) cell viability; (iv) cell concentration

Information is from the FDA website for each CTP’s Summary Basis for Regulatory Action (SBRA) [14] plus six additional sources [15–20]

*ARSA* arylsulfatase A, *CAR T-cell* chimeric antigen receptor T-cell, *CFU* colony forming unit, *HSCs* hematopoietic stem cells, *IFNγ* interferon-gamma, *MAGE4* melanoma-associated antigen A4, *MSC* mesenchymal stromal cells, *PAP-GM-CSF* human prostatic acid phosphatase-human granulocyte–macrophage colony-stimulating factor, *SBRA* FDA Summary Basis for Regulatory Action, *VCN* vector copy number

### Methods for analyzing the data

The gathered potency test information was analyzed by binning the tests by type of measurement. An example of the analysis is shown in Figure S1 for the CTP Tecartus. Figure S1 shows an excerpt from the Tecartus SBRA that lists the lot release specifications. The table shows that three measurements were used as potency tests for Tecartus: (i) “cell viability”; (ii) “anti-CD19 CAR (chimeric antigen receptor) expression” and (iii) redacted [indicated by “(b) (4)”]. “Redacted” means that the content has been censored, likely for proprietary reasons. This information was gathered for each of the 31 CTPs, organized into a spreadsheet (Supplemental File 1) and analyzed to yield the data and figures presented herein.

This analysis focuses on measurements designated as *potency tests for release testing for approved CTPs*. Tests that are not specifically listed for release testing as potency tests are not included in the analysis. There are many other categories of attributes that may be assessed by release testing such as those shown in Figure S1 for Tecartus: “appearance,” “identity,” “dose” and “safety”. Additional attribute categories that appear in SBRAs for other CTPs include “purity” and “stability” [21]. Additionally, many tests may be conducted during development and manufacturing that are not considered release tests, may not appear in the SBRA and are not the focus of this analysis. Finally, the designation of a test as a release test potency test was not made by the authors. This decision is made by the sponsor and the FDA and reported on the FDA website.

Binning is an inherently subjective process where tests that have many similarities, but also may have some differences, are grouped into categories. Binning suffers from information loss and has the potential for bias. However, simplifying complex data into discrete bins makes it easier to visualize, analyze and draw conclusions and generalizations for learning. Binning, though imperfect, helps simplify data for enhancing pattern recognition so that useful lessons can be gleaned to help make future decisions.

## Potency tests used for the 28 US FDA-approved CTPs

### Number of potency tests per CTP

**Fig. 1:**
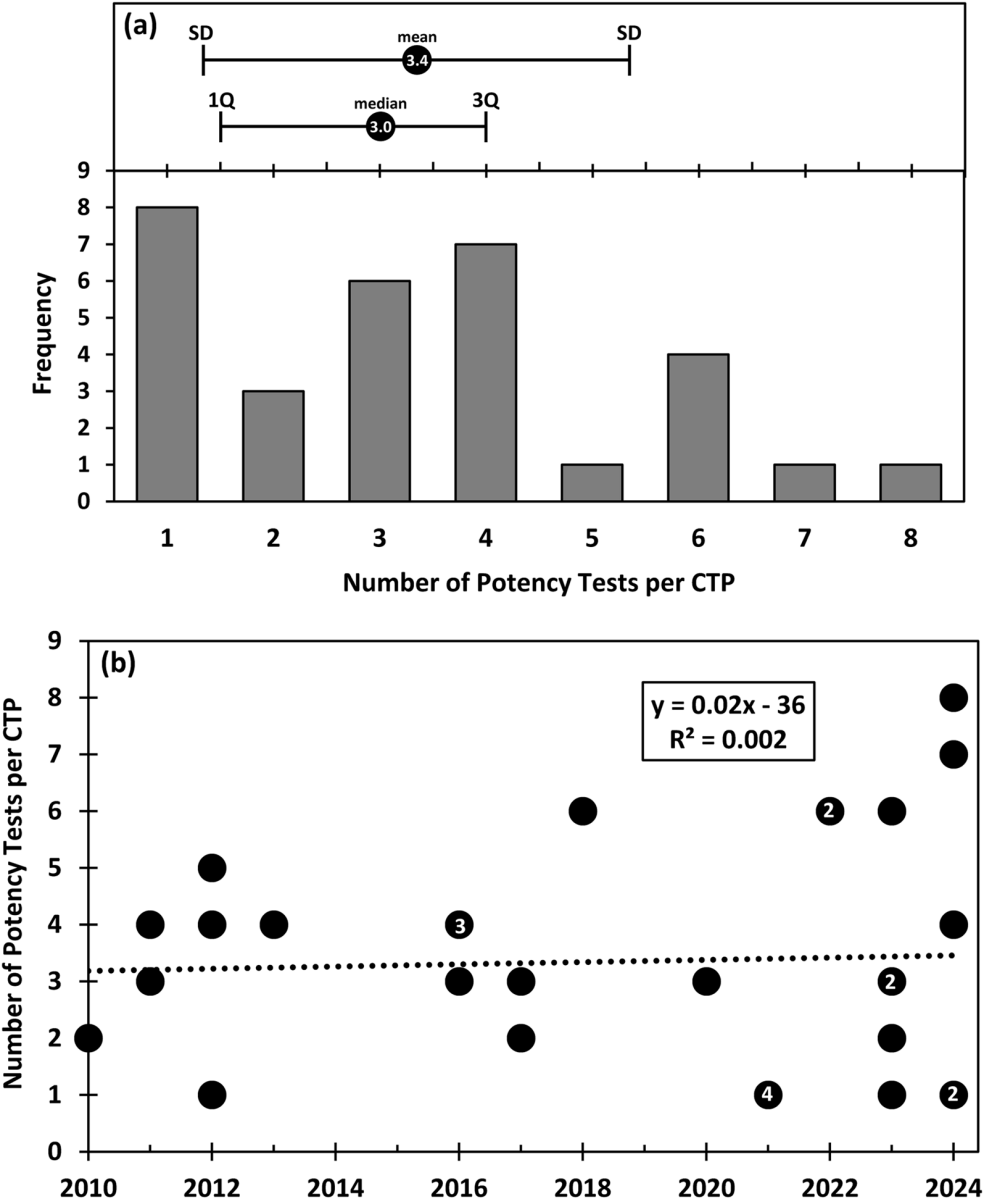
The average number of potency tests per approved CTP has remained essentially static over time (2010 to 2024). **a** Histogram showing the number of potency tests per CTP (n = 31 products). At the top of the histogram is a plot of the mean and standard deviation [mean (SD) = 3.4 (2.0)], as well as the median with first and third quartiles [median (1Q, 3Q) = 3.0 (1.5, 4.0)]. **b** Plot showing the number of potency tests per product by year. For overlapping data points, the white numbers inside the data points indicate the count of overlapping points. A linear fit to the data (dotted line) has an r^2^ of 0.002 (square of the Pearson Correlation Coefficient). Analysis of variance for linear regression had a p-value of 0.80 for the slope, indicating that the slope is not significantly different from zero

A total of 104 potency tests are reported by the manufacturers for the 31 CTPs. Figure 1a shows a histogram for the number of potency tests per CTP. The average number of potency tests per CTP is 3.4 (2.0 standard deviation) and the median is 3.0 (first quartile 1.5, third quartile 4.0). The highest number of tests for a single CTP is 8 (Lenmeldy), while 8 CTPs report only one test.

It is important to note that these numbers may not be complete, as some potency test information for the 31 CTPs is proprietary and is not disclosed (redacted). Of the 104 reported potency tests, 33 (32%) are redacted. However, information for 71 of the potency tests (68%) is available. In light of the importance of potency tests, we ought to try to learn everything that we can from the available information. Additionally, some redacted potency tests may be completely omitted from the regulatory documentation, potentially increasing the total number of potency tests beyond 104. Given these uncertainties, the mean and median number of potency tests per CTP are unlikely to be lower than shown in Fig. 1a, but they could be higher.

The data were assessed to see if there has been an increase in the number of potency tests per product over time. One might expect the number of potency tests per CTP to increase over time as science has advanced. Further, there has been emphasis on the use of a potency test matrix, which implies that more potency tests may be better than fewer potency tests [12, 13, 21, 22]. Figure 1b shows the analysis of the number of potency tests per CTP over time (plotted by year), which did not reveal a significant trend. A linear fit has a flat slope (r^2^ = 0.002) and statistical testing (linear regression analysis of variance) found that the slope is not significantly different from zero (p-value = 0.80). This is useful information for manufacturers when deciding how many potency tests to develop for new CTPs.

Table 2 shows the number of potency tests per CTP for several classes of CTPs. The hematopoietic stem cell-cord blood products had the highest number of potency tests per CTP at 4.4 (standard deviation 0.7), while the 5 tissue engineered CTPs had the lowest at 1.8 (standard deviation 1.1). The 7 CAR T-cell products also had a relatively low number of potency test per CTP at 1.9 (standard deviation 0.9).

**Table 2:** Number of Potency Tests per CTP Stratified by Product Type for the 31 US FDA-Approved CTPs

Type of CTP	CTPs	Average (SD)	Number of CTPs
Hematopoietic stem cell-cord blood	Hemacord, Clinimmune, Ducord, Lifesouth, Bloodworks, Allocord, Clevecord, MD Anderson	4.4 (0.7)	8
CAR T-cells	Kymriah, Yescarta, Tecartus, Breyanzi, Abecma, Carvykti, Aucatzyl	1.9 (0.9)	7
Tissue-engineered	Gintuit, MACI, Stratagraft, Rethymic, Lantidra	1.8 (1.1)	5
Allogeneic	Hemacord, Clinimmune, Ducord, Lifesouth, Bloodworks, Allocord, Clevecord, MD Anderson, Gintuit, Stratagraft, Rethymic, Omisirge, Lantidra, Ryoncil	3.3 (1.6)	14
Autologous	Kymriah, Yescarta, Tecartus, Breyanzi, Abecma, Carvykti, Aucatzyl, Provenge, Laviv, MACI, Zynteglo, Skysona, Casgevy, Lyfgenia, Amtagvi, Lenmeldy, Tecelra	3.4 (2.3)	17
Genetically-modified	Kymriah, Yescarta, Tecartus, Breyanzi, Abecma, Carvykti, Aucatzyl, Zynteglo, Skysona, Casgevy, Lyfgenia, Lenmeldy, Tecelra	3.3 (2.4)	13
Non-genetically-modified	Hemacord, Clinimmune, Ducord, Lifesouth, Bloodworks, Allocord, Clevecord, MD Anderson, Provenge, Laviv, Gintuit, MACI, Stratagraft, Rethymic, Omisirge, Lantidra, Amtagvi, Ryoncil	3.4 (1.7)	18
Blood-derived	Hemacord, Clinimmune, Ducord, Lifesouth, Bloodworks, Allocord, Clevecord, MD Anderson, Kymriah, Yescarta, Tecartus, Breyanzi, Abecma, Carvykti, Aucatzyl, Provenge, Zynteglo, Skysona, Omisirge, Casgevy, Lyfgenia, Amtagvi, Lenmeldy, Tecelra	3.7 (2.1)	24
Non-blood-derived	Laviv, Gintuit, MACI, Stratagraft, Rethymic, Lantidra, Ryoncil	2.3 (1.3)	7

### Types of measurements that have been used as potency tests for CTPs

#### Binning process

The 104 potency tests reported for the 31 CTPs (Table 1) were sorted into 6 bins based on perceived similarities. The 6 bins include 5 types of measurements, (i) “Viability and count”, (ii) “Expression”, (iii) “Bioassay”, (iv) “Genetic modification”, (v) “Histology” and (vi) “Redacted” (Tables 3, 4, 5, 6, 7, 8). For each potency test, the SBRA provides a short description, typically 3–20 words. In most cases, specific details of the potency test measurements are not provided and most of the available information fits into a single table (Table 1).

**Table 3:** Compilation of “Viability & Count” Potency Tests for the 31 US FDA-Approved CTPs

Product	Viability & Count	Number (Total = 37)
Hemacord (HSCs)	• “Total nucleated cells (TNC)” • (0.5) “Viability of CD45 + cells” • (0.5) “Viable CD34 + cell count” • “Colony forming unit (CFU)”	3
Clinimmune (HSCs)	• “Total nucleated cells (TNC)” • “Viability of total nucleated cells (TNC)” • (0.5) “Viable CD34 + cell count”	2.5
Ducord (HSCs)	• “Total nucleated cells (TNC)” • “Viable nucleated cells” • (0.5) “Viable CD34 + cells (flow cytometry)”	2.5
Lifesouth (HSCs)	• “Total nucleated cells (TNC)” • “Viable nucleated cells” • (0.5) “Viable CD34 + cells (flow cytometry)”	2.5
Bloodworks (HSCs)	• “Total nucleated cells (TNC)” • “Viable nucleated cells” • (0.5) “Viable CD34 + cells (flow cytometry)”	2.5
Allocord (HSCs)	• “Total nucleated cells (TNC)” • “Viable nucleated cells” • (0.5) “Viable CD34 + cell count” • “Colony forming units (CFU)”	3.5
Clevecord (HSCs)	• “Total nucleated cell number” • “Viability of TNC” • (0.5) “Viable CD34 + cell count”	2.5
MD Anderson (HSCs)	• (0.5) “Total CD34 + count” • Total nucleated cell (TNC) count (per HPC, cord blood) • “Nucleated RBC” • “Viability of nucleated cells” • (0.5) “Viable CD34 + cells” • “Colony forming unit (CFU) assay”	5
Yescarta	• “Cell viability”	1
Tecartus	• “Cell viability”	1
Carvykti	• (0.5) “CAR expression from viable T cells”	0.5
Provenge	• (0.5) “Number of CD54 + cells (flow cytometry)”	0.5
Laviv	• “Cell count” • “Cell viability”	2
MACI	• “Cell number”	1
Zynteglo	• “Colony forming cells (CFC)”	1
Lantidra	• Islet Yield: Dithizone (DTZ) stain and microscopic quantification • Viability: SYTO 13 green/ethidium bromide staining and microscopic evaluation	2
Amtagvi	• “Dose (total viable cells)”	1
Lenmeldy	• “Viability (%)”	1
Ryoncil	• “Cell viability” • “Cell concentration”	2

Quotes are from the FDA website (the product’s Summary Basis for Regulatory Action)

“(0.5)” indicates measurements that were scored as half “Viability & Count” and half “Expression”

*CAR* Chimeric antigen receptor

**Table 4:** Compilation of “Expression” Potency Tests for the 31 US FDA-Approved CTPs

Product	Expression	Number (Total = 19)
Hemacord(HSCs)	• (0.5) “Viability of CD45 + cells” • (0.5) “Viable CD34 + cell count”	1
Clinimmune(HSCs)	• (0.5) “Viable CD34 + cell count”	0.5
Ducord(HSCs)	• (0.5) “Viable CD34 + cells (flow cytometry)”	0.5
Lifesouth(HSCs)	• (0.5) “Viable CD34 + cells (flow cytometry)”	0.5
Bloodworks(HSCs)	• (0.5) “Viable CD34 + cells (flow cytometry)”	0.5
Allocord(HSCs)	• (0.5) “Viable CD34 + cell count”	0.5
Clevecord(HSCs)	• (0.5) “Viable CD34 + cell count”	0.5
MD Anderson(HSCs)	• (0.5) “Total CD34 + count” • (0.5) “Viable CD34 + cells”	1
Kymriah	• CAR expression by flow cytometry (Novartis slides from Advisory Committee Meeting)	1
Yescarta	• “Anti-CD19 CAR expression”	1
Tecartus	• “Anti-CD19 CAR expression”	1
Carvykti	• (0.5) “CAR expression from viable T cells”	0.5
Provenge	• (0.5) “Number of CD54 + cells (flow cytometry)”	0.5
Laviv	• “Collagen production by the cells”	1
MACI	• PCR measurement of aggrecan gene expression (Rapko et al., 2007)	1
Zynteglo	• “Percent LVV + cells” • “βA-T87Q-globin quantitative protein expression”	2
Skysona	• “Percent LVV + cells” • “Percent ALDP + cells”	2
Omisirge	• “CD34 + cell fold-increase”	1
Lyfgenia	• “βA-T87Q-globin quantitative protein expression”	1
Lenmeldy	• “Transduction efficiency” • “Transgene function (arylsulfatase A (ARSA) activity)”	2

Quotes are from the FDA website (the product’s Summary Basis for Regulatory Action)

“(0.5)” indicates measurements that were scored as half “Viability & Count” and half “Expression”

*ALDP* adrenoleukodystrophy protein, *CAR* chimeric antigen receptor, *LVV* lentiviral vector, *PCR* polymerase chain reaction

**Table 5:** Compilation of “Bioassay” Potency Tests for the 31 US FDA-Approved CTPs

Product	Bioassay	Score (Total = 7)
Kymriah	• “Release of IFNγ in response to CD19-expressing target cells” (Novartis slides from Advisory Committee Meeting)	1
Yescarta	• Interferon-γ production by product upon stimulation with CD19 + cells (Papadouli et al., 2020)	1
Abecma	• Interferon-γ production by product upon stimulation with BCMA + cells (EMA Assessment Report)	1
Provenge	• “Increased expression of CD54 on the surface of antigen presenting cells after culture with PAP-GM-CSF (flow cytometry)”	1
Lantidra	• “Glucose Stimulation Index (GSI): ELISA quantification of insulin release by glucose stimulated islets” (slides from Advisory Committee Meeting)	1
Tecelra	• “Cytotoxic activity (cytotoxicity assay with flow cytometry)”	1
Ryoncil	• “Interleukin-2 receptor alpha (IL-2Rα) inhibition bioassay”	1

Quotes are from the FDA website (the product’s Summary Basis for Regulatory Action)

*BCMA* B cell maturation antigen, *ELISA* enzyme linked immunosorbent assay, *IFNγ* interferon gamma, *PAP-GM-CSF* human prostatic acid phosphatase (PAP), an antigen expressed in prostate cancer tissue, linked to human granulocyte–macrophage colony-stimulating factor (GM-CSF), an immune cell activator

**Table 6:** Compilation of “Genetic Modification” Potency Tests for the 31 US FDA-Approved CTPs

Product	Genetic modification	Number (Total = 6)
Zynteglo	•”Vector copy number (VCN) (qPCR)”	1
Skysona	• “Vector copy number (VCN) (qPCR)”	1
Casgevy	• “On-target editing frequency (TIDE)”	1
Lyfgenia	• “Vector copy number (VCN)”	1
Lenmeldy	• “Vector copy number • “Vector copy number (calculation)”	2

Quotes are from the FDA website (the product’s Summary Basis for Regulatory Action)

*qPCR* quantitative polymerase chain reaction, *TIDE* Tracking of Indels by DEcomposition

**Table 7:** Compilation of “Histology” Potency Tests for the 31 US FDA-Approved CTPs

Product	Histology	Number (Total = 2)
Gintuit	• “Histology assay”	1
Rethymic	• “Histology-based potency test method”	1

Quotes are from the FDA website (the product’s Summary Basis for Regulatory Action)

There are 12 potency tests that fit into both the “Viability and count” and “Expression” bins. For example, the FDA SBRA for Carvykti cites “CAR expression from viable T cells” as a potency test. This is likely a test that measures both cell viability and CAR expression in a single measurement result (likely a flow cytometry measurement). For binning, this potency test was split between two bins where a half point (0.5) was placed in the “Viability and count” bin and a half point (0.5) was placed in the “Expression” bin. The advantage of using this half-point-system is that it does not inflate the total number of potency tests in the analysis. If a test were placed in two bins without using the half-point-system, then it would be counted twice, which would increase the total number of potency tests in the analysis and invalidate the percentage and pie chart analyses.

The binning process was carefully discussed amongst the authors to select the most accurate and honest way to represent the data. Binning is a subjective process that could be done in a variety of different ways. Many of the potency tests could be placed in more than one bin. Most tests were placed in a single bin that was determined to be the best fit. The only tests for which the half-point-system was used was *flow cytometry tests that simultaneously assessed both an expression marker and viability or count*. These tests seemed to perfectly straddle the “Viability and count” and “Expression” bins, which is why the half-point-system was reluctantly adopted for these 12 tests.

Another important point is that any given measurement might be used for a variety of applications, which complicates the binning process. A good example is flow cytometry. Flow cytometry could be used to assess (1) safety (checking for cell surface markers of cells that could be harmful), (2) purity (checking for the surface marker of the desirable cell type), (3) potency (see potency test for Provenge), (4) viability (7-aminoactinomycin D (7-AAD) staining), (5) cell count or (6) efficacy (assessing blood of the patient for decreased presence of tumor cells).

**Fig. 2:**
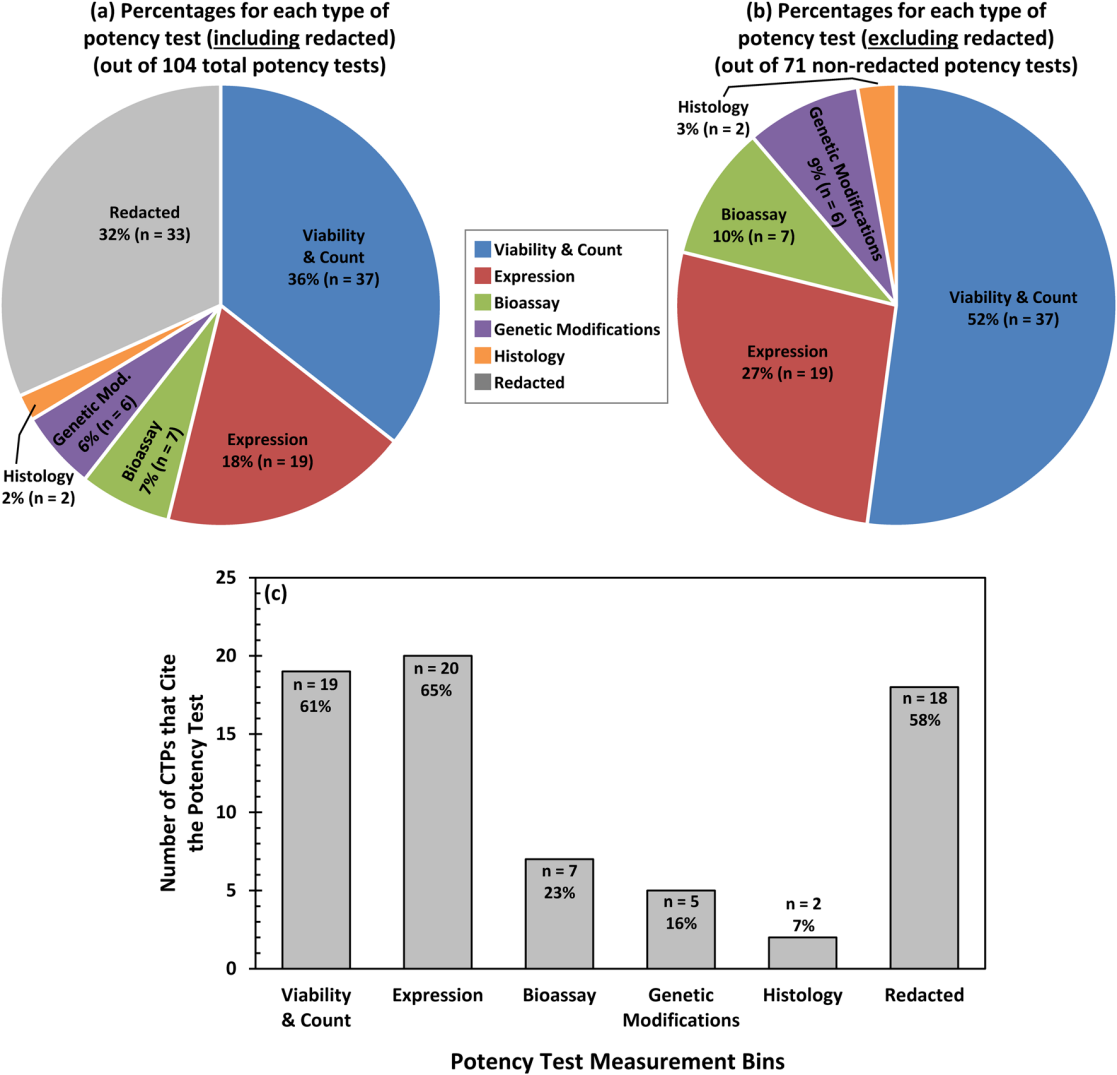
Percentages of the types of potency tests from the 31 US FDA-approved CTPs. **a** Pie chart illustrating the percentages for each type of potency test measurement (out of 104 total potency tests, including redacted tests). **b** Pie chart illustrating the percentages for non-redacted potency test measurements (out of 71 non-redacted potency tests). In both (**a**) and (**b**), the number and percentage for each test are provided. Note that “Redacted” tests are included in (**a**) but omitted in (**b**). **c** Bar graph depicting the number of CTPs (out of 31) that cite the indicated potency test measurement. Each bar shows the number and percentage (out of 31). Note that the percentages in (**c**) do not sum to 100%, since each CTP can have multiple tests

The usage frequency for each type of potency test measurement is shown in pie charts in Fig. 2. Figure 2a includes the 33 redacted potency tests while Fig. 2b omits them. The bar graph in Fig. 2c shows the number and percentage of CTPs that cite each type of potency test measurement.

#### Viability and count

The largest bin contains measurements of cell “Viability and count” with 37 potency tests as listed in Table 3 (Fig. 2). Specific details for the viability and counting measurements are not provided in the SBRA. The SBRAs provide a brief potency test description that is usually only a few words, such as “viable nucleated cells”, “cell viability”, “cell count” or “cell number” (Table 3). The SBRA does not typically specify how cell viability is measured, such as by trypan blue haemocytometer counting under a microscope, trypan blue counting in an automated imaging counter, acridine orange-propidium iodide counting in an automated fluorescence imaging counter, an MTT [3-(4,5-dimethylthiazol-2-yl)-2,5-diphenyltetrazolium bromide] plate-reader assay or staining by 7-aminoactinomycin D (7-AAD) with flow cytometry. Lantidra is an exception, since the FDA Advisory Committee Meeting Slides state “SYTO 13 green/ethidium bromide staining and microscopic evaluation” [16].

Many of the SBRA entries for potency tests have measurements such as “Viable CD34 + cell count”, which is listed for all 8 of the hematopoietic stem cell therapies (Hemacord, Clinimmune, Ducord, Lifesouth, Bloodworks, Allocord, Clevecord and MD Anderson) (Table 3). “Viable CD34 + cell count” is likely to be a flow cytometry measurement that uses a cell viability stain (such as 7-AAD) and an antibody for cell surface expression of CD34 protein [23]. For binning, “Viable CD34 + cell count” was placed as half a point in “Viability and count” and half a point in “Expression” (reflected in Table 3 and Table 4 by the following notation: “(0.5)”).

**Table 8:** Compilation of “Redacted” Potency Tests for the 31 US FDA-Approved CTPs

Product	Number redacted (Total = 33)
Clinimmune (HSCs)	1
Ducord (HSCs)	2
Lifesouth (HSCs)	1
Bloodworks (HSCs)	1
Clevecord (HSCs)	1
Tecartus	1
Breyanzi	1
Carvykti	1
Aucatzyl	1
MACI	1
Stratagraft	1
Zynteglo	2
Skysona	3
Casgevy	2
Lyfgenia	4
Amtagvi	6
Lenmeldy	3
Ryoncil	1

“Colony forming unit” (CFU) assay was also challenging to bin. CFU is cited for 4 CTPs (Hemacord, Allocord, MD Anderson, Zynteglo) and measures the number of cells that form quantifiable cell colonies. CFU was binned in “Viability and count” since it is commonly used to count the number of stem cells and progenitor cells in a cell preparation [24]. An ASTM standard was used to help make the CFU binning decision: ASTM F2944—Standard Practice for Automated Colony Forming Unit (CFU) Assays—Image Acquisition and Analysis Method for Enumerating and Characterizing Cells and Colonies in Culture. There may be a functional component to the CFU assay which signals that it could be classified as a “Bioassay”. However, the primary principle of CFU assay is cell proliferation, which, in our opinion, is too generic of a cell function to be considered a “Bioassay”.

#### Expression

“Expression” is the second largest bin with 19 potency tests as listed in Table 4 (Fig. 2). “Expression” includes measurements of molecular expression of mRNA or protein. Examples could include flow cytometry measurements for expression of cell surface markers, polymerase chain reaction (PCR) measurements of mRNA expression, enzyme-linked immunosorbent assay (ELISA) measurements of protein expression, enzyme-linked immunosorbent spot (ELISpot) measurements of protein expression and enzyme assays to measure enzyme expression. “Flow cytometry” is specifically mentioned for 5 potency tests that were binned as an “Expression” measurement (Ducord, Lifesouth, Bloodworks, Kymriah, Provenge).

However, not all flow cytometry measurements are binned under “Expression”. For example, “Flow cytometry” is cited in a potency test for Provenge and Tecelra, but in these cases it was binned in “Bioassay” as discussed in the next section. “PCR” is specifically mentioned for measuring aggrecan “Expression” for MACI [19]. Enzyme-linked immunosorbent assay (ELISA) is not specifically mentioned for measurements of protein “Expression”, but is specifically mentioned for the Lantidra “Bioassay” (Glucose Stimulation Index, discussed below) [16].

Lenmeldy’s SBRA states “transgene function (arylsulfatase A (ARSA) activity)” and was binned under “Expression” (not “Bioassay”), since it appears to be an enzymatic assay intended to measure ARSA expression [25]. Typically, an enzyme assay measures the amount of enzyme activity, which can differ from protein expression levels, especially when the enzyme is initially expressed as an inactive precursor. However, for the purpose of the binning process, which is admittedly imperfect, the ARSA enzyme assay was binned under “Expression”.

#### Bioassay

The “Bioassay” bin comprises 7% of reported potency tests (7 tests out of 104) as listed in in Table 5 (Kymriah, Yescarta, Abecma, Provenge, Lantidra, Tecelra, Ryoncil) (Fig. 2). The number could be higher since 33 potency tests are redacted. “Bioassay” is especially notable because it is emphasized in the two FDA CTP potency guidances [12, 13]. The FDA guidances describe a bioassay as an assay that has a “living biological system,” which may be cells, tissue, organ or animal [12]. The bioassay may either measure “the effect of a test article on” a living biological system or “measure the biological activity of the living cells or tissues in the product itself” [13].

For binning purposes, a bioassay was further defined as a measurement that requires live cells or tissue to respond to a stimulus. This requires a dynamic response where the behavior of the cell or tissue changes after the stimulus is applied, and this change in behavior is measured. Thus, measurement of aggrecan mRNA expression by MACI is not binned as a “Bioassay”, since MACI intrinsically expresses aggrecan. The measurement process does not include applying a stimulus to MACI that causes a change in MACI aggrecan expression levels. Likewise, measurement of collagen expression by Laviv is not binned as a “Bioassay”, since Laviv intrinsically expresses collagen. The measurement process does not include applying a stimulus to Laviv that causes a change in collagen expression levels.

Provenge, approved by US FDA for marketing in 2010, is the first CTP to cite a bioassay as a potency test: “Increased expression of CD54 on the surface of antigen presenting cells after culture with PAP-GM-CSF (flow cytometry)” [14]. For this bioassay, the CTP is the “antigen presenting cells”, which express CD54 on their cell surface following exposure to PAP-GM-CSF. PAP-GM-CSF is a recombinant fusion protein that links human prostatic acid phosphatase (PAP), an antigen expressed in prostate cancer tissue, to human granulocyte–macrophage colony-stimulating factor (GM-CSF), a cytokine and immune cell activator. To perform the potency test, the CTP is exposed to the agonist (PAP-GM-CSF) and the CTP is evaluated for increased surface expression of CD54 by flow cytometry.

Three of the CAR T-cell therapies cite an interferon-γ (IFNγ) bioassay as a potency test. For Kymriah, the Novartis slides from the FDA Advisory Committee Meeting cite “Release of IFNγ in response to CD19-expressing target cells” [15]. For this assay, the CTP is exposed to cells that express the target antigen (CD19) and the release of IFNγ is measured (presumably by ELISA). Yescarta cites the same IFNγ bioassay as Kymriah [17]. Abecma cites a similar IFNγ bioassay, except that the cells express a different target antigen, B cell maturation antigen (BCMA), instead of CD19 [18].

The Lantidra FDA Advisory Committee Meeting slides cite the fifth bioassay: “Glucose Stimulation Index (GSI): ELISA quantification of insulin release by glucose stimulated islets” [16]. For this measurement, the Lantidra CTP is challenged with glucose and release of insulin is measured by ELISA.

Tecelra SBRA cites “Cytotoxic activity (cytotoxicity assay with flow cytometry)” as a potency test. Although the details of this test are unknown, it may involve adding the CTP to target cells expressing human melanoma-associated antigen (MAGE-A4) and then measuring a response, such as the percentage of target cells that are killed or T-cell degranulation measured by assessing the exposure of CD107a on the surface of T-cells [26].

Ryoncil SBRA cites “interleukin-2 receptor alpha (IL-2Rα) inhibition bioassay” as a potency test. Details of the assay have not been disclosed. However, it was binned as a bioassay because the FDA document entitled “Clinical and Clinical Pharmacology Review and Evaluation” specifically used the word “bioassay” to describe this potency test [20]. The Ryoncil clinical trial report states that “graft versus host-disease therapy with mesenchymal stems cells is associated with reductions in relevant inflammatory biomarkers, including IL-2Rα,” and that the cells “suppress IL-2Rα expression on activated lymphocytes” [27].

The potency test for Omisirge is cited as “CD34 + cell fold-increase” in the SBRA (Table 1). This sounds like it could be a “Bioassay”, but not enough information is available to make this determination. Thus, it was binned as “Expression”.

#### Genetic modification

The bin for measurements of “Genetic modification” has 6 potency tests as listed in Table 6 (Fig. 2). These tests are for genetically modified CTPs. “Genetic modification” is a separate bin from “Expression” since measuring a genetic modification is not the same as measuring the expression of mRNA or protein. A “Genetic modification” is a change to a cell’s DNA sequence that is intended to cause a change in expression of mRNA or protein. A separate bin for “Genetic modification” was created since many CTPs have been genetically modified and because “Genetic modification” measurements have emerged as their own class of potency tests.

“Genetic modification” includes two types of measurements. The first is “vector copy number (VCN)”, which can be a PCR amplification of genomic DNA to count the average number of copies of a gene that are present in a cell preparation. A VCN of 1 or greater may confirm the presence of a genetic modification made to the CTP [28]. Four CTPs cite VCN as a potency test: Zynteglo, Skysona, Lyfegnia and Lenmeldy (with two VCN potency tests cited for Lenmeldy). VCN is often considered a “safety” test instead of a “potency” test. When VCN is used as a potency test, a VCN of 0 would mean that the CTP had not be genetically modified and could not achieve its intended mechanism of action [28]. When VCN is used as a safety test, a high copy number could be considered unsafe since it may increase the risk of insertional mutagenesis [29].

The second measurement is the TIDE assay which is cited for Casgevy as “On-target editing frequency (TIDE)”. TIDE stands for “Tracking of Indels by Decomposition” and is an assay that compares PCR-amplified genomic DNA from the CTP to the unmodified cells to assess for the presence of gene edits [30].

#### Histology

**Fig. 3:**
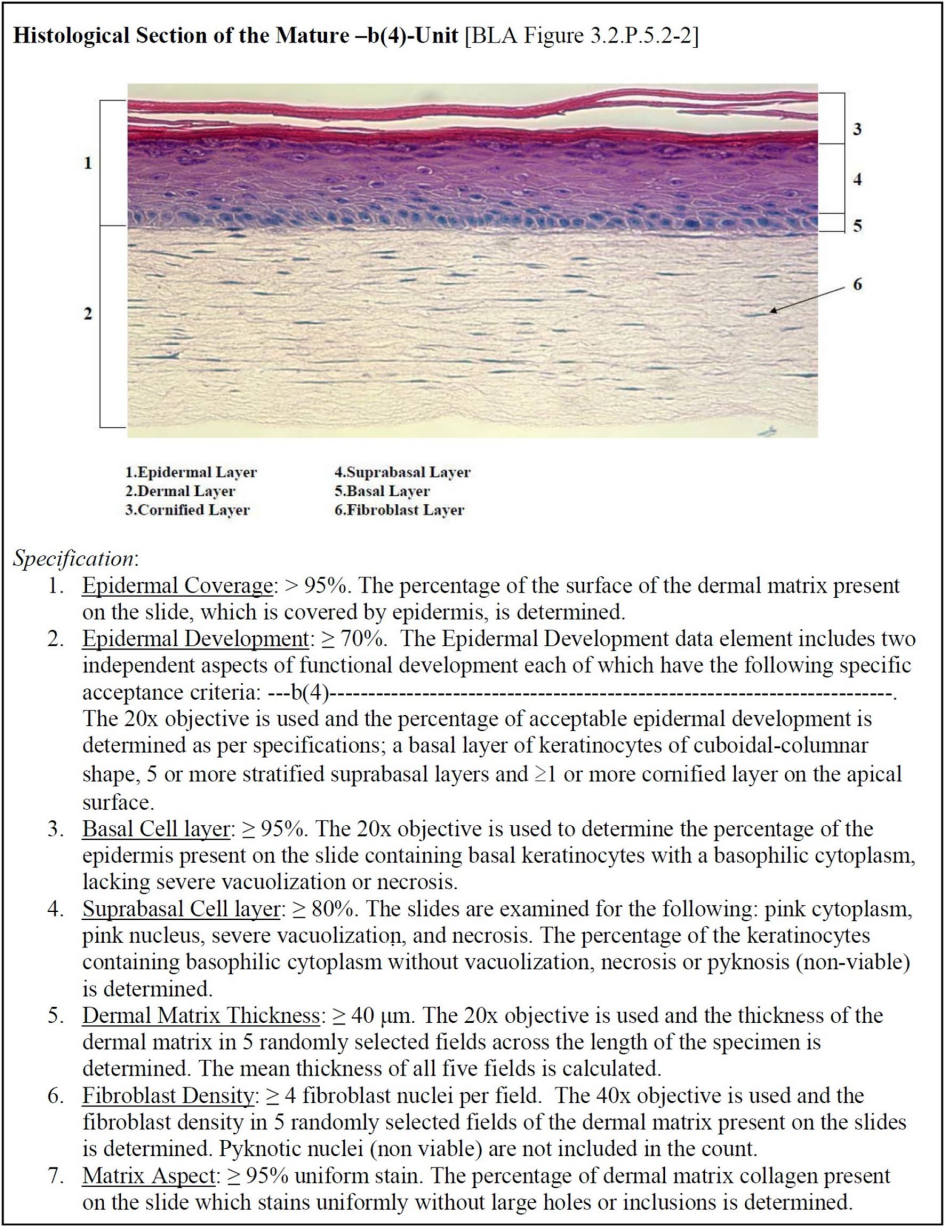
Gintuit potency test: histological measurements. The content of the figure is an excerpt from the FDA Briefing Document for the FDA Gintuit Advisory Committee Meeting that was held on November 17, 2011 [31]. The image at the top shows a micrograph of histological section of biopsy punch of a manufactured unit of Gintuit. The biopsy punch was fixed, embedded, sectioned, stained by hematoxylin and eosin and imaged. The histological sections are assessed for the 7 structural parameters listed in the bottom part of the figure

The “Histology” bin includes two tissue engineered products, Gintuit and Rethymic (Table 7, Fig. 2). Details of the Rethymic histology potency test are not available, but the Gintuit FDA documentation provides more information about the Gintuit potency test than does any of the other FDA documentation for any of the other CTP potency tests [31, 32]. The test involves taking a punch biopsy from a manufactured unit of the product followed by fixing, embedding, sectioning, staining by hematoxylin and eosin and microscopy exam to assess the structural properties. “Product potency is determined by a set of histological parameters which collectively assess the quality of the epidermal and dermal layers present in the product after maturation. These parameters include epidermal coverage, epidermal development, basal cell layer, suprabasal cell layer, dermal matrix thickness, fibroblast density, and matrix aspect.” Fig. 3 shows an excerpt from a US FDA document with a representative histological section from Gintuit along with a description of 7 structural features that are measured.

Of note, Gintuit evolved from an older CTP called Apligraf, which was approved for marketing by the US FDA in 1998 [33]. A potency test was not required for Apligraf since it was regulated as a “medical device” (instead of as a “biologic”). The US FDA does not require a potency test for medical device approvals, but requires a potency test for biologics approvals. Also of note is the SBRA for Stratagraft, allogeneic cultured keratinocytes and dermal fibroblasts in murine collage for wound closure in deep thickness burns. The Stratagraft SBRA lists “histology” as a “lot release test” but not as a “potency test”.

#### Number of CTPs that cite each type of potency test measurement

The number of CTPs that cite each type of potency test measurement are plotted in Fig. 2c. Measurements of “Expression” are the most widely used potency test and were cited by 20 of the 31 CTPs (65%). Cell “Viability and count” measurements were also common being cited by 19 CTPs (61%). Eighteen CTPs (58%) had potency tests that were “Redacted”. In addition, five CTPs (16%) cited measurements of “Genetic modifications” and two (7%) cited “Histology” as potency tests.

“Bioassay” was saved for last since these tests are a major focus of FDA potency guidances [12, 13]. Due to the large number of redactions, it is unclear how many CTPs use bioassays are as potency tests. “Bioassay” was cited by 7 CTPs (23%), but the number could be higher due to redactions. There are another 17 CTPs that have redacted tests which could be bioassays. If all 17 had a bioassay, then this would bring the total number of CTPs with a bioassay potency test to 24, which would be 77% of CTPs. As a CTP developer, it may be useful to know what is not known. Thought leaders frequently emphasize the importance of “knowing what you don’t know” (known unknowns) versus “not knowing what you don’t know” (unknown unknowns). Awareness of what is not known is of substantial value.

Note that the data only reflect tests that were used for release testing as potency tests. For example, 19 of the 31 CTPs cite “Viability and count” as a potency test, which does not mean that the 12 other CTPs were not assessed for viability or count. This only means that one of the potency tests was a measurement of “Viability and count”. If “Viability and count” were measured during development, manufacturing or as another type of release test, such as dose, safety or purity, then this would not be captured by our analysis.

### Upset plot to show how potency tests are used together

**Fig. 4:**
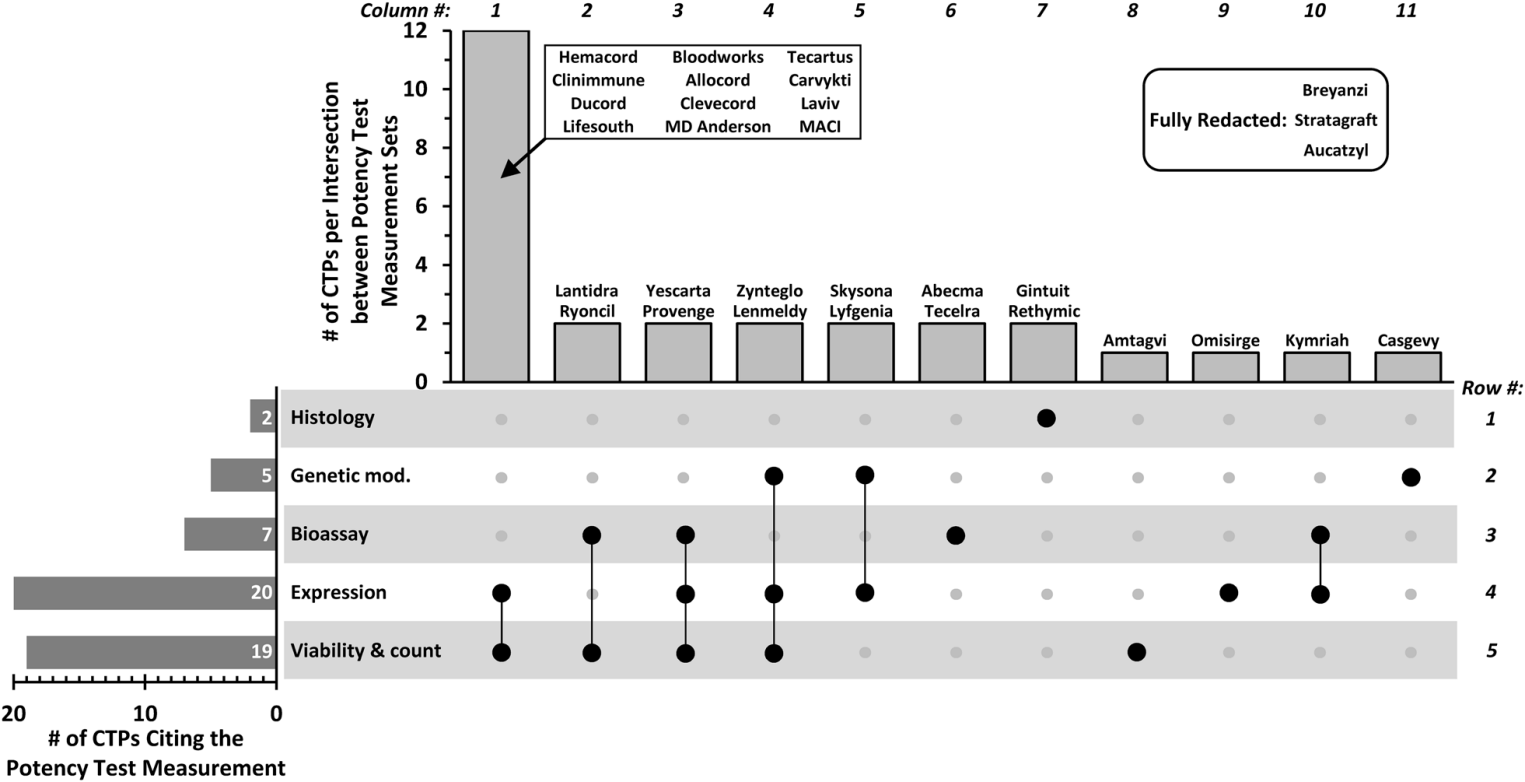
Intersections between potency test measurement sets: “Viability & count” and “Expression” are used together the most (even more than any single measurement alone). The figure is an “Upset Plot” which serves the same purpose as a Venn diagram but is easier to depict for 5 sets. The bar graph at the bottom left shows the size of each set: the number of CTPs that cite each of the measurements as a potency test. The black circles and vertical lines in bottom table indicate intersections between sets. The top bar graph displays the number of CTPs that fall at each intersection. Example 1: The two black circles connected by a bar to the right of “Expression” and “Viability & count” (column 1, rows 4 and 5) identifies the twelve CTPs that cite only these two types of potency tests. Example 2, the single black circle in column 7, row 1 identifies the two CTPs (Gintuit and Rethymic) that cite “Histology” as the only non-redacted potency test. The upset plot includes 28 CTPs (not 31) since potency tests for three CTPs were fully redacted

An analysis of how the 5 types of potency test measurements are used together for individual CTPs can be informative (Fig. 4). Similar to Venn diagrams, “upset plots” are a way to visualize relationships between multiple sets.

- The upset plot in Fig. 4 shows one strong trend: “Viability and count” and “Expression” are the two types of potency tests most commonly used together for the same CTP. They are used together as potency tests for 16 of 31 CTPs (52%; sum of columns 1, 3 and 4; Fig. 4). For 12 CTPs, they are the only non-redacted potency tests (column 1, Fig. 4).
- “Expression” and “Genetic modification” measurements are cited together as potency tests for 4 CTPs (Zynteglo, Lenmeldy, Skysona, Lyfgenia) (columns 4 and 5, Fig. 4). For Skysona and Lyfgenia, they are the only non-redacted potency tests (column 5, Fig. 4).
- “Viability and count” and “Bioassay” measurements are cited together as potency tests for 4 CTPs (Lantidra, Ryoncil, Yescarta, Provenge,) (columns 2 and 3, Fig. 4). For Lantidra and Ryoncil, they are the only non-redacted types of potency tests (column 2, Fig. 4).
- “Expression” and “Bioassay” measurements are cited together as potency tests for 3 CTPs (Yescarta, Provenge, Kymriah) (columns 3 and 10, Fig. 4). For Kymriah, they are the only non-redacted potency tests (column 10, Fig. 4).
- Two CTPs (Yescarta, Provenge) cite only the following 3 types of measurements as potency tests: “Viability & count”, “Expression” and “Bioassay” (column 3, Fig. 4).
- Two CTPs (Zynteglo, Lenmeldy) cite only the following 3 types of measurements as potency tests: “Viability & count”, “Expression” and “Genetic modification” (column 4, Fig. 4).

### Trends of measurements used as potency tests over time

**Fig. 5:**
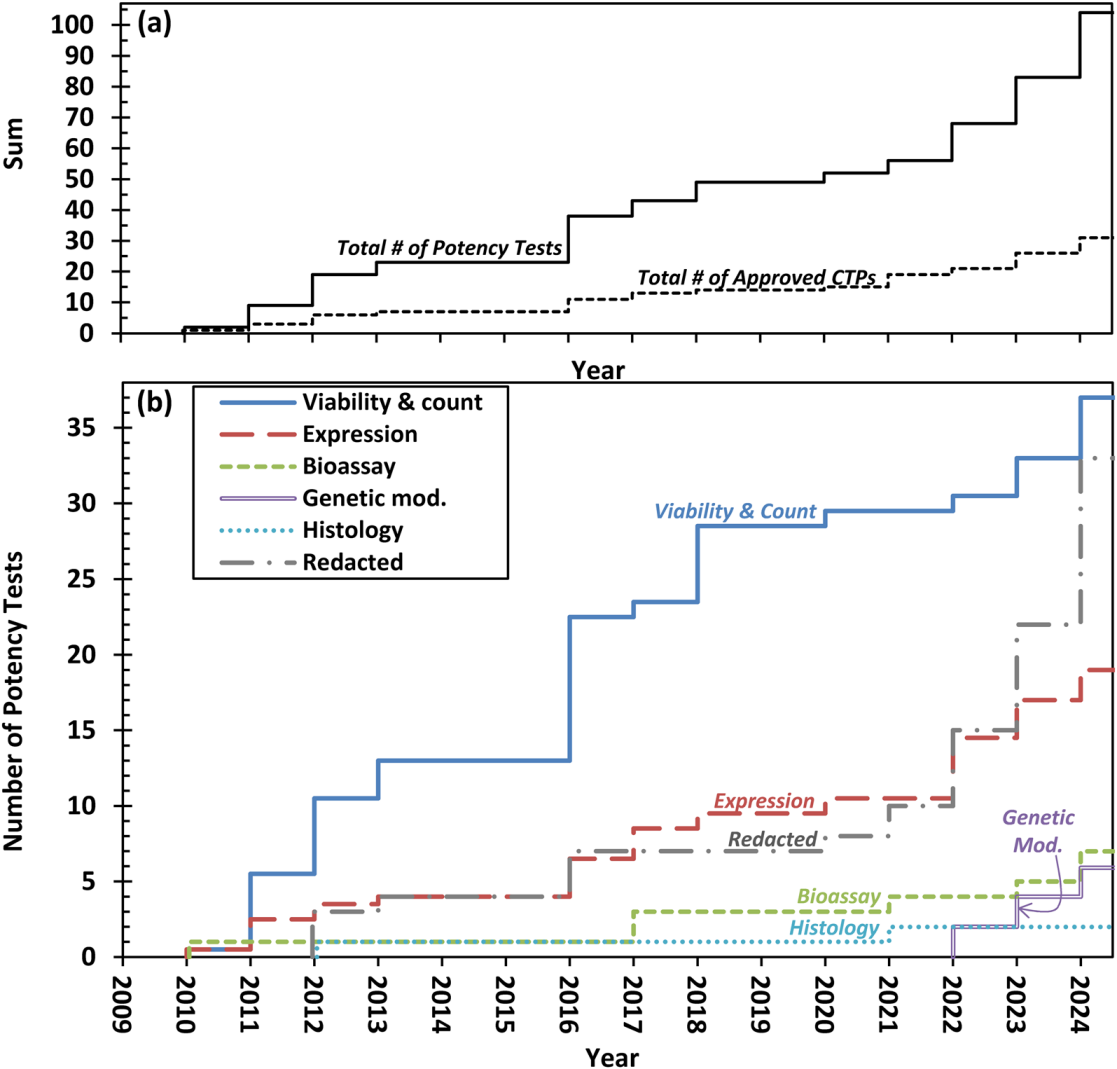
Trends in measurements used as potency tests over time. **a** Cumulative sum step graph showing (i) the total number of approved CTPs by year and (ii) total number of potency tests by year since 2010. **b** Cumulative sum step graph showing the total number of potency tests for each type of measurement by year

Figure 5 presents a cumulative sum step graph illustrating the trends in the types of measurements used as potency tests for the 31 CTPs. Three notable trends are evident. First, “Viability and count” and “Expression” measurements have been used consistently since 2010 when Provenge was first approved.

Second, “Genetic modification” measurements are relatively new as potency tests, first appearing with the approval of Zynteglo in 2022. This does not mean that “Genetic modification” tests such as VCN or TIDE, are new. VCN has been used for testing CTPs for many years. Instead, this analysis indicates that *use of VCN for release testing as a “potency test” for approval of a CTP* is new.

Third, Potency test redactions seem to be on the rise. There were only 15 potency test redactions for 21 CTPs between 2010 and 2022 (0.7 redactions per CTP), but there were 18 redactions for 10 CTPs in 2023 and 2024 (1.6 redactions per CTP). Thus, 55% (18/33) of potency test redactions occurred in 2023 and 2024.

## CTP potency test measurements discussed in US FDA guidances

There are 5 FDA guidance documents [12, 13, 34–36] and one FDA Town Hall [37] that provide relevant information on measurements that may be suitable as CTP potency tests (Table 9). Guidance documents represent the current thinking of the US FDA on a topic and are not binding to the FDA, CTP developers or the public. Following is a comparison between ‘the potency tests discussed in the US FDA guidances’ and ‘the potency tests reported for the 31 US FDA-approved CTPs’. Note that the numbers below could be higher since an estimated 32% of potency tests (33 of 104) are redacted.

**Table 9:** CTP Potency Test Measurements Discussed in US FDA Guidances and Town Halls

US FDA guidance	Type of CTP addressed	Potency test measurements discussed
Guidance for Industry—Potency Tests for Cellular & Gene Therapy Products (2011) [12]	Cell therapies, general	• “Bioassays” (“performed in a living system”) • “Non-biological analytical assay”: If development of a bioassay is not feasible, then “it may be necessary to identify a surrogate measurement of biological activity” (“performed outside of a living system”) • “Immunochemical” properties: “quantitative flow cytometry, enzyme-linked immunosorbent assay” • “Molecular” properties: “reverse transcription polymerase chain reaction, quantitative polymerase chain reaction, microarray” • “Biochemical” properties: “protein binding, enzymatic reactions”
Guidance for Industry—Biologics License Applications for Minimally Manipulated, Unrelated Allogeneic Placental/Umbilical Cord Blood Intended for Hematopoietic and Immunologic Reconstitution in Patients with Disorders Affecting the Hematopoietic System (2014) [34]	Allogeneic hematopoietic stem cell therapies (HSCs) (cord blood)	• “Total nucleated cells (TNC)” • “Viable nucleated cells” • “Viable CD34 + cells (flow cytometry)”
Draft Guidance for Industry—Potency Assurance for Cellular and Gene Therapy Products (2023) [13]	Cell therapies, general	• “Bioassays”: Assays that measure “the effect of a test article on living cells, tissues, or animals” or “a biological activity of the living cells or tissues in the product itself” • “Physicochemical assays”: “assays that are not bioassays”
Guidance for Industry—Considerations for the Development of Chimeric Antigen Receptor (CAR) T Cell Products (2024) [35]	Chimeric antigen receptor T cell products (CAR T-cells)	• “Flow cytometry” • “Cytokine secretion assays” • “Transduction efficiency measurements” • “Cell killing assay”
Guidance for Industry–Human Gene Therapy Products Incorporating Human Genome Editing (2024) [36]	Ex vivo-modified gene-edited cell therapies	• "Test confirming the desired genetic sequence modification" • "Assessment of the intended downstream biological modification (e.g., corrected cellular function)”
OTP Town Hall: Cell Therapy CMC Readiness for Late-Stage INDs (2024) [37]	Tissue-engineered medical products	• “Biomolecular markers, biochemical properties, immunological responses, biomechanical strength and other relevant factors that are mechanistically linked to the product’s biological activity.” • “When cells are seeded into or onto a scaffold, its crucial to assess the integrity of the final construct and the distribution of cells within the scaffold. Ensuring uniform cell distribution and proper structural integrity is essential for the product’s overall potency and functionality.”

*Quotes indicate language quoted from US FDA guidance or FDA Town Hall recordings*

### US FDA 2011 and 2023 potency guidances

The US FDA has two generalized guidances on CTP potency tests (Table 9). The first was published in 2011 [12] and a second (in draft stage) was published in 2023 [13]. Both guidances emphasize using a bioassay as a potency test. Bioassays are defined as a measure of potency conducted “within a living biological system” [12]. Further, a bioassay is defined as an assay that measures “the effect of a test article on living cells, tissues, or animals” or “a biological activity of the living cells or tissues in the product itself” [13]. The true number of CTPS that use bioassays is not known. Only 7 of the 31 CTPs (23%) report a “Bioassay” as a potency test (Fig. 2c, Table 5) but the number could be much higher due to redactions.

The 2011 guidance also discusses use of “non-biological analytical assays” “in cases where development of a suitable bioassay is not feasible” [12]. The 2011 guidance defines “non-biological analytical assay” as assays “performed outside of a living system” citing examples such as flow cytometry, ELISA, PCR and enzymatic reactions [12] (Table 9). The 2023 draft guidance also allows for the use of non-bioassays but uses a different term to describe them, “physicochemical assays”, which are defined as “assays that are not bioassays” [13].

- 26 of the 31 CTPs (84%) cite physicochemical assays (non-bioassays) as potency tests.
- The only 5 CTPs that do not cite a physicochemical assay are 1) Breyanzi, 2) Aucatzyl, and 3) Stratagraft, whose potency tests are fully redacted; and 3) Abecma and 4) Tecelra, which each cite only 1 potency test, a “Bioassay”.

Both the 2011 and 2023 potency guidances also suggest that it may be necessary to have multiple potency tests (referred to as a potency “assay matrix”), since “a single biological or analytical assay may not provide an adequate measure of potency” [12, 13].

- Twenty three of the 31 US FDA-approved CTPs (76%) cite more than one potency test (Table 1).
- The 8 CTPs that cite only a single potency test are Omisrige (“Expression”), Breyanzi (redacted), Abecma (“Bioassay”), Aucatzyl (redacted), Gintuit (“Histology”), Stratagraft (redacted), Rethymic (“Histology”) and Tecelra (“Bioassay”).

### US FDA 2014 cord blood CTP guidance

The US FDA has a 2014 guidance on allogeneic cord blood CTPs intended for hematopoietic and immunologic reconstitution in patients with disorders affecting the hematopoietic system [34]. The 2014 guidance recommends 3 potency tests for these CTPs: (i) “total nucleated cells (TNC)”; (ii) “viable nucleated cells”; and (iii) “viable CD34 + cells (flow cytometry)”. The guidance also gives recommended specifications for these potency tests.

- Of the 9 allogeneic cord blood CTPs, all but Omsirge cite “total nucleated cells (TNC)” and “viable CD34 + cells” as potency tests (Table 1).
- All but Hemacord and Omsirge cite “viable nucleated cells” as a potency test.

### US FDA 2024 CAR T-cell therapy guidance

The US FDA issued a 2024 guidance on chimeric antigen receptor (CAR) T-cell products [35]. This guidance specifically mentions 4 measurements that may be suitable as potency tests for CAR T-cell therapies: (i) “flow cytometry”, (ii) “cytokine secretion assays”, (iii) “transduction efficiency measurements” and (iv) “cell killing assay”.

- Of the 7 FDA-approved CAR T-cell therapies, only Kymriah (“CAR expression by flow cytometry”) specifically cites “flow cytometry” (Table 1).
- Three of the CAR T-cell therapies (Kymriah, Yescarta, Abecma) cite a cytokine secretion assay as a potency test (release of IFNγ in response to antigen-expressing cells).
- None of the CAR T-cell therapies cite a “cell-killing assay” or “transduction efficiency measurements” as a potency test (Table 1).
- “Transduction efficiency measurements” are mentioned as a potency test for one CTP, Lenmeldy, which is not a CAR T-cell therapy.
- Amtagvi is a T-cell therapy (though not a CAR T-cell therapy) which has 7 potency tests. Six are redacted and one is binned as “Viability & count” (“dose (total viable cells)”).
- Tecelra is a T-cell receptor T-cell therapy (TCR-T), which is similar to a CAR T-cell therapy. Tecelra cites a “cell killing assay” (“cytotoxic activity”) as its only potency test.

### US FDA 2024 gene therapy product guidance

The US FDA also released a guidance on “Human Gene Therapy Products Incorporating Human Genome Editing” in 2024 [36]. This guidance has information for “ex vivo-modified gene-edited cell therapies” and recommends two types of potency tests: (i) “test confirming the desired genetic sequence modification” and (ii) “assessment of the intended downstream biological modification (e.g., corrected cellular function)”. For (ii), the language in the guidance focuses on cell function, suggesting more than a measurement of mRNA or protein “Expression”, and seems to imply “Bioassay”. Casgevy is the only US FDA-approved CTP that is gene-edited and it is edited with CRISPR/Cas9 (clustered regularly interspaced short palindromic repeats/CRISPR-associated protein 9). However, there are 13 genetically modified CTPs (Kymriah, Yescarta, Tecartus, Breyanzi, Abecma, Carvykti, Zynteglo, Skysona, Casgevy, Lyfgenia, Lenmeldy, Tecelra, Aucatzyl) which could potentially fall under the purview of this guidance.

- Five of 13 (38%) (Zynteglo, Skysona, Casgevy, Lyfgenia, Lenmeldy) cite a “test confirming the desired genetic sequence modification” as a potency test (VCN or TIDE assay) (Table 1).
- Four of 13 (31%) (Kymriah, Yescarta, Abecma, Tecelra) cite an “assessment of the intended downstream biological modification (e.g., corrected cellular function)” as a potency test (“Bioassay”) (Table 1).

### US FDA 2024 town hall on cell therapy CMC readiness

In September 2024 at a US FDA Town Hall entitled “Cell Therapy CMC (Chemistry, Manufacturing and Controls) Readiness for Late-Stage INDs (Investigational New Drug)” [37] (Table 9), one of the questions was “How does the FDA recommend assessing the potency of complex tissue-engineered products in late-stage development, especially when the product’s therapeutic effect is influenced by multiple cell types or scaffold materials?” The FDA said “These assays should evaluate key attributes like biomolecular markers, biochemical properties, immunological responses, biomechanical strength and other relevant factors that are mechanistically linked to the product’s biological activity.” The FDA also said “When cells are seeded into or onto a scaffold, its crucial to assess the integrity of the final construct and the distribution of cells within the scaffold. Ensuring uniform cell distribution and proper structural integrity is essential for the product’s overall potency and functionality.” There are 5 US FDA-approved tissue engineered CTPs: Gintuit, MACI, Stratagraft, Rethymic and Lantidra.

- One (20%) cites “biomolecular marker”: MACI cites PCR measurement of aggrecan gene expression.
- One (20%) cites “biochemical properties”: Lantidra cites dithizone staining of islets. Dithizone is a red stain for zinc granules in islet beta cells and zinc is required for proper processing, storage and function of insulin [38].
- None cite “immunological responses” or “biomechanical strength” as a potency test.
- Three (60%) (Gintuit, Rethymic, Lantidra) cite an assessment of “distribution of cells within the scaffold” or “proper structural integrity”: Gintuit (Fig. 3) and Rethymic cite “Histology” and Lantidra mentions “SYTO 13 green/ethidium bromide staining and microscopic evaluation”.

In closing, measurements that are not specifically suggested as potential CTP potency tests in the FDA guidances and town halls may be suitable as potency tests for CTPs. For example, the following measurements are not specifically discussed in the FDA guidances but are cited as potency tests for US FDA-approved CTPs: CFU, VCN, TIDE, CD34 + cell fold-increase, histology and glucose stimulation index (Table 1).

## Discussion

Determining appropriate potency tests for CTPs is challenging. The current perspective presents an analysis of the potency tests used for the 31 US FDA-approved cell therapy products. Data-driven, analytical approaches are inherently backward-looking and rest on the assumption that the past can inform the future. There is no guarantee that the measurements that are used as potency tests for the currently approved CTPs will make adequate potency tests for future CTPs.

The results presented in this paper are not absolute and should only serve as a guide, since approximately one third (32%) of the estimated 104 CTP potency tests are redacted. On the other hand, approximately two thirds of the (68%) of the potency tests have been disclosed. There is value in analyzing the information that has been disclosed to learn as much as possible about the measurements that have been used as potency tests for approved CTPs.

In the future, it would be helpful if CTP sponsors could share information about the potency tests for approved CTPs. Basic information, even if it is just a few words as shown in Table 1, would be insightful and better than a redaction. Better still would be if sponsors published papers detailing the analytical methods used for CTP approval. The authors know of one example: an analysis of the correlation between the potency test results and clinical outcome for allogeneic cord blood for immunologic reconstitution in patients undergoing myeloablative treatments to treat malignancies [39]. This example provides detailed methods for measuring cord blood total nucleated cell count, mononuclear cell count, CD34 + cell count and CFU. In contrast, cell therapy clinical trial publications typically provide detailed descriptions of how the efficacy endpoints are measured, which is extremely helpful for interpreting clinical data.

There are proprietary considerations that can prevent sharing of potency test information. However, granting agencies could potentially develop mechanisms to incentivize grantees to share potency test information and other analytical methods for new CTPs – particularly when CTPS have been developed using public funds. In reports from Phase 1 or Phase 2 clinical trials, it would be helpful if research teams could publish more information, preferably detailed analytical methods; and discuss their potency test strategy. Other than the example above [39], we are not aware of other examples where the analytical methods or potency tests for manufacturing and release are discussed for a Phase 1/2 CTP clinical trial.

Standards and reference materials (RMs) for CTP potency tests could be helpful for CTP development. There are existing standards on cell counting [40–42], measuring cell viability in scaffolds [43, 44] and characterizing cell CTPs [21, 45]. A standard guide that describes the potency tests that may be suitable for different classes of CTPs could be helpful. Standard test methods that focus on specific measurements, such as the IFNγ measurements or VCN, could also be useful. Standard test methods provide a detailed protocol describing how to perform the measurement along with data on repeatability (within lab variability) and reproducibility (between lab variability) determined via inter-laboratory testing [46, 47]. This information can help CTP developers to define appropriate targets and expectations for potency test specifications. RMs can be used for measurement validation or calibration and several relevant RMs exist. The National Institute for Biological Standards and Control (NIBSC) has RM vascular endothelial growth factor (VEGF) [48] and RM bone morphogenetic protein-2 (BMP-2) [49]. VEGF and BMP-2 have been involved in the proposed mechanisms of action for several CTPs and these RMs could be used to validate or calibrate potency tests that measure these molecules. The US Pharmacopeia offers a fixed, lyophilized CD34 + cell RM that can be used as a positive control in flow cytometry measurements [50]. Synthetic cell RMs that are hydrogels that mimic the optical properties of cells can be used as positive controls for flow cytometry measurements [51].

## Conclusions

An analysis of the measurements used as potency tests for the 31 US FDA-approved CTPs was conducted. Twenty of the 31 CTPs (65%) cited measurements of gene or protein expression and 19 (61%) cited measurements of cell viability or cell count. Notably, 16 of 31 (52%) used both “viability and count” and “expression” as potency tests. Although FDA guidances emphasize bioassays, it is unclear if bioassays are commonly used at potency tests. Only 7 of 31 CTPs (23%) reported bioassays as potency tests. However, due to redactions, this number may be as a high as 24 CTPs (77%). In addition, 26 of 31 CTPs (84%) cite physicochemical assays (non-bioassays) as potency tests. This analysis of the state of the art for potency test measurements provides valuable insights for designing potency tests for future CTPs.

## Supplementary Information

Additional file 1.

Additional file 2.

## Data Availability

The dataset supporting the conclusions of this article is included as Supplemental file 1.

## Abbreviations

7-AAD: 7-Aminoactinomycin D
ALDP: Adrenoleukodystrophy protein
ARSA: Arylsulfatase A
BCMA: B cell maturation antigen
BMP-2: Bone morphogenetic protein-2
CAR: Chimeric antigen receptor
CFU: Colony forming unit
CMC: Chemistry, manufacturing and controls
CTP: Cell therapy product
ELISA: Enzyme-linked immunosorbent assay
ELISpot: Enzyme-linked immunosorbent spot
FDA: Food and Drug Administration
HSCs: Hematopoietic stem cells
IL-2Rα: Interleukin-2 receptor alpha
IFNγ: Interferon-gamma
IND: Investigational new drug
LVV: Lentiviral vector
MAGE4: Melanoma-associated antigen A4
MSC: Mesenchymal stromal cells
MTT: 3-(4,5-Dimethylthiazol-2-yl)-2,5-diphenyltetrazolium bromide
NIBSC: National Institute for Biological Standards and Control
PAP-GM-CSF: Human prostatic acid phosphatase-human granulocyte-macrophage colony-stimulating factor
PCR: Polymerase chain reaction
RM: Reference material
SBRA: Summary Basis for Regulatory Action
TIDE: Tracking of indels by decomposition
TNC: Total nucleated cells
US: United States
VCN: Vector copy number
VEGF: Vascular endothelial growth factor
