# Supplementary material for: Analysis of the measurements used as potency tests for the 31 US FDA-approved cell therapy products
Source: J Transl Med. 2025 Mar 4;23:259. doi: 10.1186/s12967-025-06253-4 (PMC11877884; doi:10.1186/s12967-025-06253-4)
Supplement: Supplementary file 1 — Additional file 1. [file 12967_2025_6253_MOESM1_ESM.pdf]

3 potency test slots: 2 are revealed & 1 is redacted

1 viability

1 expression

1 redacted

Table 1. TECARTUS lot release specifications

Attribute	Test	Sample Point (Process Step)	Analytical Procedure	Acceptance Criteria
Appearance	Visual Appearance Inspection	Inspection and Labeling	Visual Inspection (SOP-00624)	White to red, including shades of white, light yellow, and orange. Clear to opaque liquid with no visible foreign particles
Identity	(b) (4)	Formulation	(b) (4) to detect the scFv heavy chain variable region, linker and CD28 sequences (CAR: (b) (4)	(b) (4)
Dose[1]	Viable Cell Count/Anti-CD19 CAR Expression	N/A[1]	(b) (4)	2×10^6 Anti-CD19 CAR T cells/Kg (Maximum allowable dose 2×10^8 Anti-CD19 CAR T cells based on patient weight ≥ 100 kg)
Potency	Cell Viability	(b) (4)	(b) (4)	(b) (4)
	Anti-CD19 CAR Expression	(b) (4)	(b) (4)	(b) (4)
	(b) (4)	(b) (4)	(b) (4)	(b) (4)
Safety	Mycoplasma	(b) (4)	(b) (4))	Negative
	Sterility	Formulation	(b) (4)	Negative
	Endotoxin	Formulation	(b) (4)	(b) (4)
	(b) (4)	Formulation	(b) (4)	(b) (4)

Abbreviations: CAR = chimeric antigen receptor; (b) (4) *quantitative polymerase chain reaction;* (b) (4)

[1] *Dose is calculated based on patient weight, viable cell concentration and* (b) (4) *(anti-CD19 CAR expression)*

Figure S1. Example of how the potency test data were collected and binned. The table on the right was taken from the "Summary Basis for Regulatory Action" posted on the FDA website and shows lot release specifications for Tecartus. The table indicates that Tecartus has three potency tests, one of which is redacted. "(b) (4)" indicates a redaction (i.e., information that is censored). Note that other types of lot release specifications are shown: appearance, identity, dose, safety.

Source: Summary Basis for Regulatory Action - Tecartus, FDA, 2020. Accessed February 3, 2025: https://www.fda.gov/vaccines-blood-biologics/cellular-gene-therapy-products/approved-cellular-and-gene-therapy-products
